# Identification of a novel interaction site between the large hepatitis delta antigen and clathrin that regulates the assembly of genotype III hepatitis delta virus

**DOI:** 10.1186/s12985-022-01866-3

**Published:** 2022-10-17

**Authors:** Wei-Chung Chiou, Hsu-Feng Lu, Jui-Chieh Chen, Yu-Heng Lai, Ming-Fu Chang, Yuan-Li Huang, Ni Tien, Cheng Huang

**Affiliations:** 1 Department of Biotechnology and Laboratory Science in Medicine, National Yang Ming Chiao Tung University, No. 155, Sec. 2, Linong St., Beitou District, Taipei, 11221 Taiwan; 2 Department of Medical Laboratory Science and Biotechnology, Asia University, Taichung, 41354 Taiwan; 3 Department of Laboratory Medicine, China Medical University Hospital, Taichung, 40402 Taiwan; 4 Department of Biochemical Science and Technology, National Chiayi University, Chiayi, 60004 Taiwan; 5 Department of Chemistry, Chinese Culture University, Taipei, 11114 Taiwan; 6 Institute of Biochemistry and Molecular Biology, School of Medicine, National Taiwan University, Taipei, 10051 Taiwan

**Keywords:** HDV genotypes, Clathrin heavy chain, Hepatitis delta antigen, W box, Clathrin box

## Abstract

**Background:**

Hepatitis delta virus (HDV), a satellite virus of hepatitis B virus (HBV), is a small, defective RNA virus strongly associated with the most severe form of hepatitis and progressive chronic liver disease and cirrhosis. Chronic hepatitis D, resulting from HBV/HDV coinfection, is considered to be the most severe form of viral hepatitis and affects 12–20 million people worldwide. Involved in the endocytosis and exocytosis of cellular and viral proteins, clathrin contributes to the pathogenesis and morphogenesis of HDV. Previously, we demonstrated that HDV-I and -II large hepatitis delta antigens (HDAg-L) possess a putative clathrin box that interacts with clathrin heavy chain (CHC) and supports HDV assembly.

**Methods:**

Virus assembly and vesicular trafficking of HDV virus-like particles (VLPs) were evaluated in Huh7 cells expressing HDV-I, -II and -III HDAg-L and hepatitis B surface antigen (HBsAg). To elucidate the interaction motif between HDAg-L and CHC, site-directed mutagenesis was performed to introduce mutations into HDAg-L and CHC and analyzed using coimmunoprecipitation or pull-down assays.

**Results:**

Comparable to HDV-I virus-like particles (VLPs), HDV-III VLPs were produced at a similar level and secreted into the medium via clathrin-mediated post-Golgi vesicular trafficking. Mutation at F27 or E33 of CHC abolished the binding of CHC to the C-terminus of HDV-III HDAg-L. Mutation at W207 of HDV-III HDAg-L inhibited its association with CHC and interfered with HDV-III VLP formation. We elucidated mechanism of the binding of HDV-III HDAg-L to CHC and confirmed the pivotal role of clathrin binding in the assembly of genotype III HDV.

**Conclusions:**

A novel W box which was identified at the C terminus of HDV-III HDAg-L is known to differ from the conventional clathrin box but also interacts with CHC. The novel W box of HDAg-L constitutes a new molecular target for anti-HDV-III therapeutics.

**Supplementary Information:**

The online version contains supplementary material available at 10.1186/s12985-022-01866-3.

## Introduction

Hepatitis delta virus (HDV) is a small, defective RNA virus strongly associated with the most severe form of hepatitis and progressive chronic liver disease and cirrhosis [1, 2]. Because of the coexistence of HBV, either through coinfection with both viruses or superinfection of an established HBV carrier, chronic hepatitis D, which affects 12–20 million people worldwide, is considered to be the most severe form of viral hepatitis [3, 4]. To date, eight HDV genotypes (HDV-I–VIII) have been reported. HDV-I is the predominant genotype worldwide causing a broad spectrum of symptoms, while HDV-II (formerly IIa) and HDV-IV (formerly IIb) have been reported to be prevalent in Southeast Asia, China, Japan, and Taiwan with a milder disease progression than HDV-I [5, 6]. HDV-III is the most divergent genotype, mainly located in the Northern part of South America, and has been found to often contribute to severe liver disease [2, 3].

The HDV virion comprises an inner ribonucleoprotein (RNP) structure, consisting of 70–200 hepatitis delta antigen (HDAg) proteins and a circular negative-sense single-stranded RNA (–ssRNA) genome of about 1.7 kb, and an outer envelope of HBV surface antigen (HBsAg) [2]. HDAg has two forms, the HDAg-small (HDAg-S, 195 amino acids, 24 kDa) and the HDAg-large (HDAg-L, 214 amino acids, 27 kDa) proteins. These HDAg isoforms differ by 19 or 20 amino acids (genotype dependent) at the C terminus of HDAg-L, which is the result of RNA editing of a UAG stop codon to a UGG tryptophan codon [3, 7, 8]. This difference makes some post-translational modifications (PTMs) exclusive to one or other isoform, thereby controlling the balance between viral synthesis, mediated by HDAg-S, and particle assembly, modulated by HDAg-L [3]. Several types of PTMs of HDAg-S have been described and these regulate the replication of antigenomic RNA and synthesis of genomic RNA and mRNA and govern RNA localization [2]. Similarly, PTMs increase the functional diversity of HDAg-L, including its association with HBsAg and virus particle assembly [2]. Large HBsAg, which contains a domain that binds to the cellular receptor, is known to be essential for the infectivity of HDV virions [9] but small HBsAg alone is sufficient for the production of HDV viral particles [10]. Prenylation of the cysteine residue at the C terminus of HDAg-L is integral to the formation of virus-like particles (VLPs) with small HBsAg [11–13].

Studies have shown that the N-terminal β-propeller domain of clathrin interacts with four independent sequence motifs, the clathrin box (LФXФ[D/E]), the W box (PWXXW), the arrestin box ([L/I][L/I]GXL), and the Royle box (the motif sequence is yet to be determined), where Ф is a bulky hydrophobic amino acid, X is any amino acid, and brackets enclose alternatives [14–18]. In addition, it has been demonstrated that the first 100 residues (blades 7, 1, and 2) of the N-terminal β-propeller domain of clathrin are sufficient for binding in vitro and that the groove between blades 1 and 2 is important for interaction with the adaptor molecules [19, 20]. Previously, we demonstrated that the C terminus of HDAg-L from HDV-I and HDV-II interacts with the clathrin heavy chain (CHC) to form clathrin-coated vesicles (CCVs) in the *trans*-Golgi network (TGN) and to support HDV assembly efficiently [21, 22]. Considering that the C-terminal region of HDV-III HDAg-L is highly divergent [2, 3], whether the morphogenesis of HDV-III involves an alternative interaction with CHC remains unclear.

Here, we aimed to study the assembly and secretion HDV-III by resolving the interaction between HDV-III HDAg-L and CHC. Virus assembly and vesicular trafficking were evaluated in Huh7 cells expressing HDV-III VLPs composed of the HDV-III HDAg-L and small HBsAg. Site-directed mutagenesis of HDAg-L and CHC identified the putative clathrin-binding motif at the C terminus of HDV-III HDAg-L and the residues of CHC involved. The W box located at the C terminus of HDV-III HDAg-L has a major role in clathrin-binding and the morphogenesis of HDV-III.

## Materials and methods

### Plasmids

All expression constructs were sequenced for accurate incorporation.

(i) *pECE-C-ES*. Plasmids pECE-C-ES contain cDNAs encoding small HBsAg [11].
(ii) *pCMV-Tag2C-HDAgL, pCMV-Tag2C-HDAgL-II, pCMV-Tag2C-HDAgL-III, pCMV-Tag2C-HDAgL-III-W207A, and pCMV-Tag2C-HDAgS-III*. Plasmids pCMV-Tag2C-HDAgL and pCMV-Tag2C-HDAgL-II encode full-length HDAg-L from HDV-I and HDV-II, respectively, as published previously [22]. Full-length HDV-III HDAg-L and HDAg-S cDNA sequences were inserted into the plasmid pCMV-Tag2C with additional recognition sequences of BamHI/HindIII at the ends. The HDAg-L-III-W207A mutant was generated using a site-directed mutagenesis kit (Stratagene Cat.200521, San Diego, USA) to introduce a mutation into the plasmid pCMV-Tag2C-HDAgL-III.
(iii) *pGEX-HDAg-L(198–210), pGEX-HDAg-L(198–210)-II, pGEX-HDAg-L(198–210)-III, and pGEX-HDAg-L(198–210)-III-W207A.* Plasmids pGEX-HDAg-L(198–210) and pGEX-HDAg-L(198–210)-II published previously encode GST-tagged fusion proteins GST-I(198–210) and GST-II(197–209) [22]. For the construction of plasmid pGEX-HDAg-L(198–210)-III encoding GST-HDAg-L(198–210)-III, HDV-III HDAg-L(198–210) generated by annealing two synthetic oligonucleotide strands was cloned into the vector pGEX-6P-1, restricted by EcoRI and SalI (Amersham Parmacia Biotech Ltd, Little Chalfont, UK). Derived from the plasmid pGEX-HDAg-L(198–210)-III, the plasmid pGEX-HDAg-L(198–210)-III-W207A encoding GST-HDAg-L(198–210)-III-W207A was generated using a site-directed mutagenesis kit (Stratagene Cat.200521, San Diego, USA) with the HDAg-L-III-W207A primer set: 5’-ACTCATGGGACGGCGTAATACCCGGGGGGA-3’ and 5’-TCCCCCCGGGTATTACGCCGTCCCATGAGT-3’.
(iv) *pET15b-CHC*_*1-107*_*, pET15b-CHC*_*1-107*_*-F27A, and pET15b-CHC*_*1-107*_*-E33A.* Plasmid pET15b-CHC_1-107_, published previously, encodes His-CHC(1–107), a His tag was fused with N-terminal domain of CHC from amino acids 1 to 107 [21]. His-CHC(1–107)-F27A and His-CHC(1–107)-E33A mutants were generated by site-directed mutagenesis of the pET15b-CHC_1-107_ template, using the CHC-F27A primer set: 5’-GCAAACATTGGCGCCAGTACCCTGAC-3’ and 5’-GTCAGGGTACTGGCGCCAATGTTTGC-3’, and the CHC-E33A primer set: 5’-CTGACTATGGCGTCTGACAAA-3’ and 5’-TTTGTCAGACGCCATAGTCAG-3’, respectively.
(v) *pEGFP-N1-CHC*_*1-107*_*, pEGFP-N1-CHC*_*1-107*_*-F27A, and pEGFP-N1-CHC*_*1-107*_*-E33A.* The plasmid pEGFP-N1-CHC_1-107_, published previously, encodes GFP-CHC(1–107),, the GFP protein fused to the N-terminal domain of CHC from amino acids 1 to 107 [22]. Mutations encoding F27A and E33A were introduced into the plasmid pEGFP-N1-CHC_1-107_ using a site-directed mutagenesis kit (Stratagene Cat.200521, San Diego, USA) with the CHC-F27A primer set, and the CHC-E33A primer set, respectively. The resultant plasmids pEGFP-N1-CHC_1-107_-F27A and pEGFP-N1-CHC_1-107_-E33A encode GFP-CHC(1–107)-F27A and GFP-CHC(1–107)-E33A, respectively.

### Cell line and chemicals

Huh7 cells were maintained in Dulbecco’s modified Eagle’s medium (DMEM) with 10% heat-inactivated fetal bovine serum (FBS), 100 U/mL of penicillin, 100 µg/mL of streptomycin, and 100 nM nonessential amino acids supplements (Thermo Fisher Scientific, Waltham, MS, USA).

### Harvesting of HDV virus-like particles (VLPs) and determination of packaging activity

DNA transfection was performed as described previously [23] using Lipofectamine 2000 (Thermo Fisher Scientific Cat. 11,668,019, Waltham, MS, USA). HDV VLPs were collected from the culture supernatant of Huh7 cells at 4 days posttransfection as described previously [23]. For viral packaging activity, collected VLPs were subjected to western blot analysis.

#### Antibodies, coimmunoprecipitation, and western blot analysis

Rabbit polyclonal antibodies specific to HDAg was generated as described previously [24]. Mouse monoclonal antibody against CHC (BD Biosciences, Franklin Lakes, NJ, USA), mouse monoclonal antibody against Flag epitope (Sigma-Aldrich, St. Louis, MI, USA), goat polyclonal antibodies specific to HBsAg (Aligent Technologies, Santa Clara, CA, USA), mouse monoclonal antibodies directed against GFP and His epitope (Clontech Laboratories, Mountain View, CA, USA), and horseradish peroxidase-conjugated goat anti-mouse IgG and horseradish peroxidase-conjugated goat anti-rabbit secondary antibodies (Jackson Laboratory, Bar Harbor, ME, USA) were used as suggested. Coimmunoprecipitation [25] and western blot analysis [23] were performed as described previously. Densitometry analysis was performed in FIJI [26].

#### Purification of recombinant fusion proteins and GST pull-down assay

GST, GST-tagged, and His-tagged fusion proteins were expressed in *Escherichia coli* BL21 and purified as described previously [21]. Huh7 cells were lysed in PBS containing 1% Triton X-100 and a protease inhibitor cocktail (Sigma-Aldrich Cat. P8340, St. Louis, MI, USA) and centrifuged to remove cell debris. GST fusion proteins were purified with glutathione-Sepharose 4A beads at 4 °C for overnight. The beads were precipitated, and extensively washed with PBS containing 1% Triton X-100.

##### Phylogenetic analysis of HDAg-L

From NCBI Virus database [27], 1628 accessions of HDAg-L were retrieved according to the definition of HDV HDAg-L reported previously [3], and the C-terminal 19 or 20 amino acid sequences of HDAg-L were shown in Additional File 2: Table S1. Among the 1628 HDV accessions, 217 unique C-terminal amino acid sequences of HDAg-L were identified (Additional File 3: Table S2). Data were analyzed in Matlab R2020a (The Mathworks Inc., Natick, MA, USA). Pairwise distances between the unique C-terminus of HDAg-L were calculated using the default seqpdist function. The phylogenetic tree was constructed with the neighbor joining method using the default seqneighjoin function. Clustering was performed using the cluster function with the maxclust set to 3. The sequence logos of sequences were produced using the default seqlogo function.

#### Statistical analysis

Data (N = 3) are shown as the mean ± SEM. Data were analyzed in GraphPad Prism 7.0 (GraphPad Software, San Diego, CA, USA). For statistical significance compared with the control, one-way ANOVA with Dunnett’s multiple comparisons tests were performed (*p < 0.05; **p < 0.01; ***p < 0.001).

## Results

### The efficiency of assembly and secretion of HDV-III is comparable to HDV-I

**Fig. 1:**
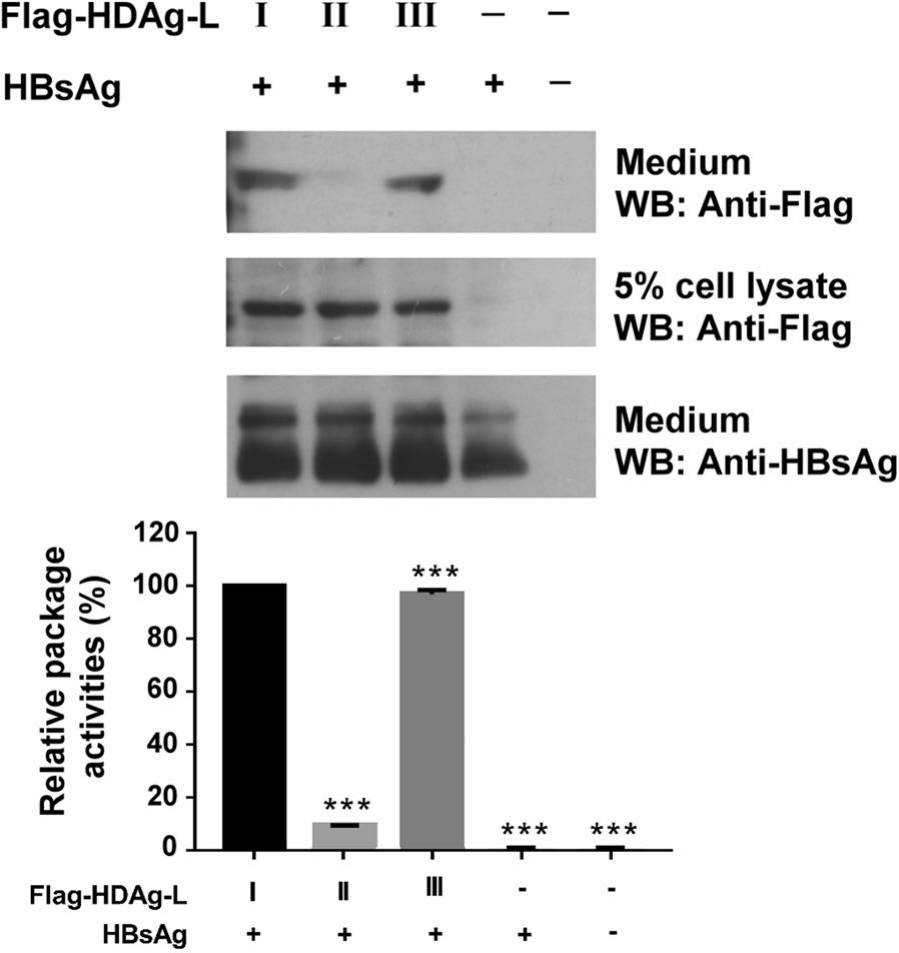
Production of HDV-I, HDV-II and HDV-III VLPs. Secretion of HDV-I, HDV-II, and HDV-III VLPs from Huh7 cells in the presence of small HBsAg. HDV VLPs were harvested from the medium of Huh7 cells cotransfected with pECE-C-ES and pCMV-Tag2C-HDAgL, pCMV-Tag2C-HDAgL-II, or pCMV-Tag2C-HDAgL-III at day 4 posttransfection. Levels of Flag-tagged HDAg-L and small HBsAg were detected by western blot analysis using anti-Flag and anti-HBsAg antibodies. Experiments were performed in triplicate. Results of western blots were quantified by densitometry analysis. Protein quantification is shown in the bottom panel

The assembly of HDV-I VLPs is more efficient than that of HDV-II VLPs [22]. To compare the assembly efficiency of HDV-III with HDV-I and HDV-II, Huh7 cells were cotransfected with plasmids encoding HBsAg and HDV-I, HDV-II, or HDV-III HDAg-L. The HDV-I and HDV-III VLPs were secreted into the medium efficiently by the transfected Huh7 cells, but the secretion of HDV-II VLPs was less efficient (Fig. 1). The amounts of HDV-I, HDV-II, or HDV-III HDAg-L in the cell lysates and small HBsAg in the medium were comparable between the transfectants. Taken together, in the presence of small HBsAg, HDV-I and HDV-III VLPs were assembled and secreted more efficiently than HDV-II VLPs.

### HDV-III HDAg-L is a novel clathrin binding protein

**Fig. 2:**
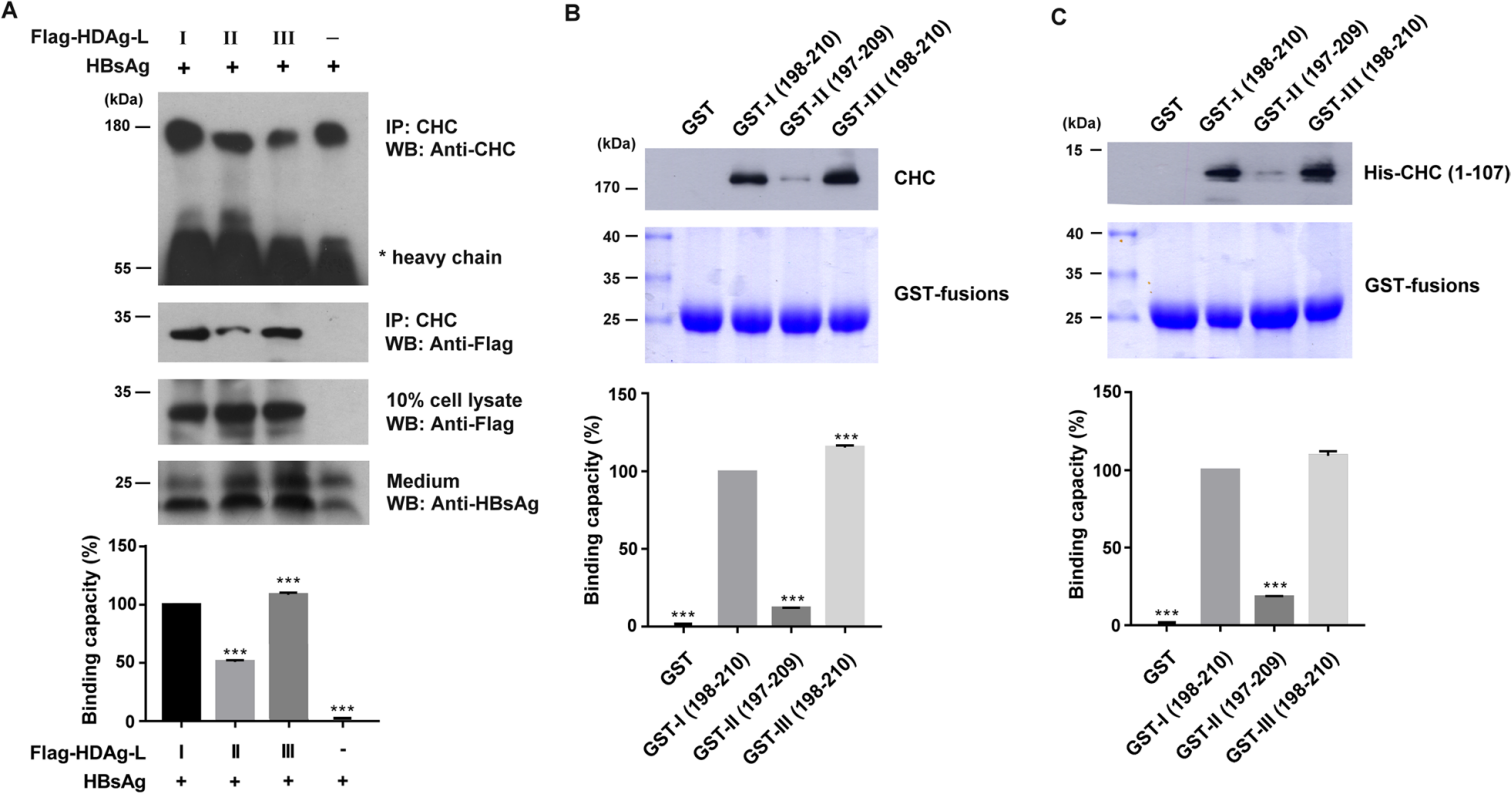
Binding of HDV-I, HDV-II, and HDV-III HDAg-L to CHC.** A** Coimmunoprecipitation of CHC with Flag-tagged HDAg-L. Lysates of Huh7 cells cotransfected with plasmids encoding HBsAg and HDV-I, HDV-II, or HDV-III HDAg-L were incubated with the anti-CHC antibody for precipitation. Levels of CHC, Flag-tagged HDAg-L, and small HBsAg were determined by western blot analysis by using anti-CHC, anti-Flag and anti-HBsAg antibodies. **B** Pull-down of endogenous CHC with GST-tagged C-terminal HDAg-L. GST-tagged proteins were incubated with the Huh7 cell lysates. Levels of CHC were determined by western blot analysis by using anti-CHC antibody. Coomassie blue staining of GST fusion proteins is shown in the bottom panel. **C** Pull-down of His-CHC(1–107) with GST-tagged C-terminal HDAg-L. GST-tagged proteins were incubated with the purified recombinant His-CHC(1–107). Levels of His-CHC(1–107) were determined by western blot analysis using anti-His antibody. Coomassie blue staining of GST fusion proteins is shown in the bottom panel. Experiments were performed in triplicate. Results of western blots were quantified by densitometry analysis

HDV-I and -II HDAg-L have been reported to interact with CHC [21, 22], so that we investigated whether HDV-III HDAg-L binds to CHC. Coimmunoprecipitation assays were performed using Huh7 cell producing HDV-I, HDV-II, or HDV-III VLPs. As shown in Fig. 2A, coimmunoprecipitation of HDAg-L with CHC revealed that CHC interacted with HDV-I and HDV-III HDAg-L more strongly than with HDV-II HDAg-L, with little effect on the expression level of CHC. The amounts of HDAg-L in the cell lysates and small HBsAg in the medium were comparable between the transfectants. Similar results were observed in COS7 cells (Additional File 1: Fig. S1). The N-terminal domain of CHC, from amino acids 1–107, has been reported to be responsible for binding to HDV-I and -II HDAg-L [21, 22]. To determine whether HDV-III HDAg-L interacts directly with CHC, GST pull-down assays were performed using GST-tagged proteins containing the C terminus of HDV-I HDAg-L from amino acids 198–210 (GST-I(198–210)), HDV-II HDAg-L from amino acids 197–209 (GST-II(197–209)), and HDV-III HDAg-L from amino acids 198–210 (GST-III(198–210)). Later, GST-tagged proteins were incubated with the Huh7 cell lysate or the purified recombinant His-CHC(1–107). As shown in Fig. 2B, GST-I(198–210) and GST-III(198–210) captured more endogenous CHC from the Huh7 cell lysate than did GST-II(197–209). Similarly, the recombinant His-CHC(1–107) interacted with GST-I(198–210) and GST-III(198–210) more strongly than with GST-II(197–209) (Fig. 2C). The level of binding of CHC to the C terminus of HDV-III HDAg-L was akin to that of HDV-I HDAg-L. Taken together, our data confirm HDV-III HDAg-L to be a novel clathrin binding protein.

### F27 and E33 play critical roles in the binding of CHC to HDV-III HDAg-L and facilitate HDV-III morphogenesis

**Fig. 3:**
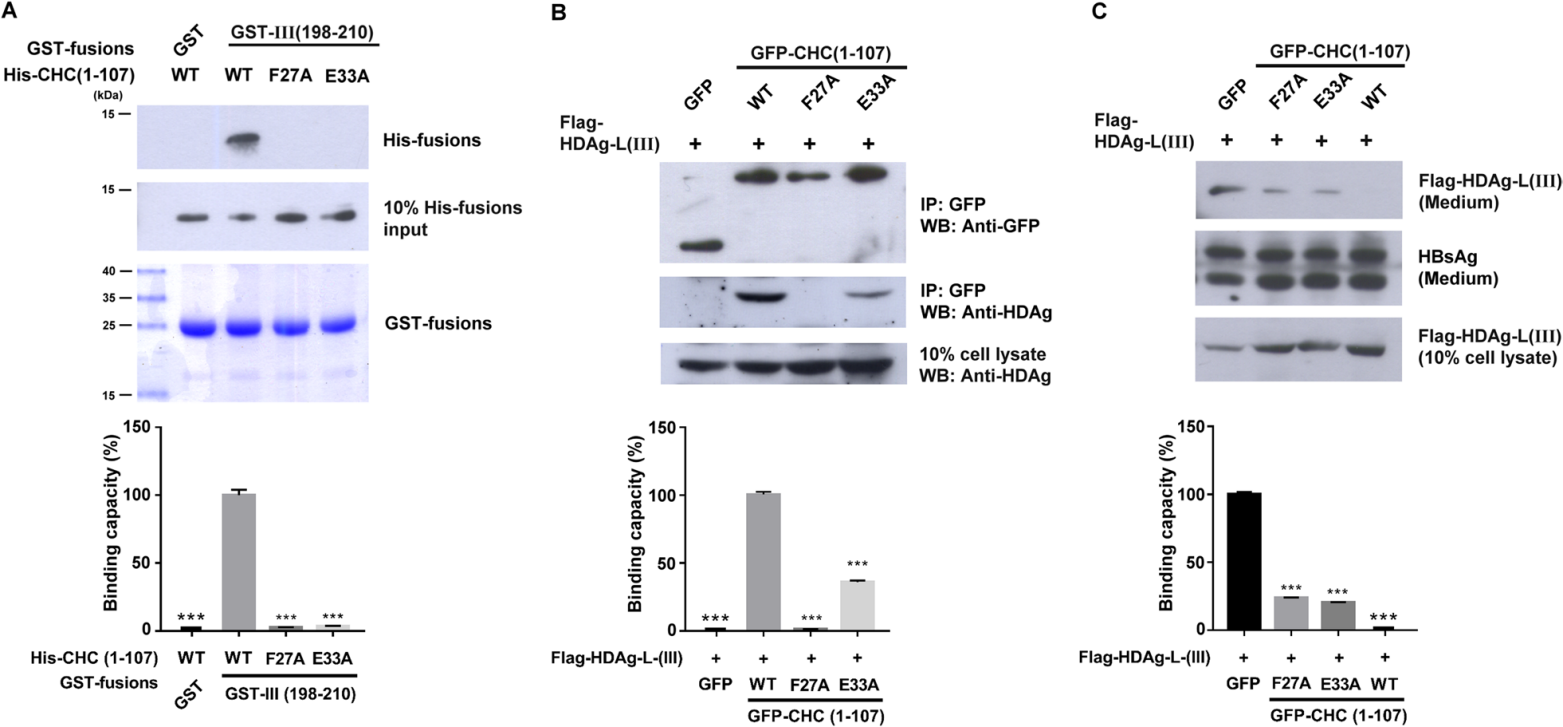
The role of F27 and E33 in CHC for HDV-III VLP formation.** A** Binding of GST-III(198–210) to His-CHC(1–107) and its mutants His-CHC(1–107)-F27A, or His-CHC(1–107)-E33A. GST-tagged proteins were incubated with His fusion proteins. Levels of His fusion proteins were determined by western blot analysis using anti-His antibody. Coomassie blue staining of GST fusion proteins is shown in the bottom panel. **B** Coimmunoprecipitation of GFP-CHC(1-107) and its mutants GFP-CHC(1-107)-F27A, or GFP-CHC(1-107)-E33A with HDV-III HDAg-L. At day 2 posttransfection, lysates of Huh7 cells cotransfected with plasmids pCMV-Tag2C-HDAgL-III and pEGFP-N1-CHC_1-107_, pEGFP-N1-CHC_1-107_-F27A, or pEGFP-N1-CHC_1-107_-E33A were incubated with the anti-GFP antibody for precipitation. Levels of GFP fusion proteins and HDV-III HDAg-L were determined by western blot analysis using anti-GFP antibody and anti-HDAg antibodies. **C** Secretion of HDV-III VLPs in the presence of GFP-CHC(1-107) and its mutants GFP-CHC(1-107)-F27A and GFP-CHC(1-107)-E33A. HDV-III VLPs were harvested from the medium of Huh7 cells cotransfected with plasmids pCMV-Tag2C-HDAgL-III and pEGFP-N1-CHC_1-107_, pEGFP-N1-CHC_1-107_-F27A, or pEGFP-N1-CHC_1-107_-E33A at day 4 posttransfection. Levels of Flag fusion proteins and small HBsAg were determined by western blot analysis. Experiments were performed in triplicate. Results of western blots were quantified by densitometry analysis

Previously, we reported that CHC is important for HDV assembly by mediating post-Golgi membrane trafficking in the cytoplasm [22]. To determine the residues at the N terminus of CHC responsible for binding to HDV-III HDAg-L, site-directed mutagenesis was carried out on the plasmid pET15b*-*CHC_1-107_ to generate His-tagged CHC(1–107)-F27A and CHC(1–107)-E33A mutants. As shown in Fig. 3A, when the purified His-CHC(1–107) and GST-tagged proteins were at a comparable level, the GST-III(198–210) bound to wild type CHC(1–107), but neither mutant was able to form interactions with the GST-III(198–210). In parallel, two mutants, GFP-tagged CHC(1–107)-F27A and CHC(1–107)-E33A, were constructed using the plasmid pEGFP-N1-CHC_1-107_ as a template and transfected into Huh7 cells for protein expression. The interactions between wild type CHC(1–107) and the two mutants with HDV-III HDAg-L in Huh7 cells were investigated by co-immunoprecipitation analysis. Coimmunoprecipitation of GFP-CHC(1–107) with HDV-III HDAg-L showed that the F27A and E33A substitutions abolished the binding of CHC(1–107) to HDV-III HDAg-L (Fig. 3B). Meanwhile, GFP alone cannot form a complex with HDV-III HDAg-L. Previously, we showed that CHC(1–107) acts as a dominant-negative mutant that competes with full-length CHC for HDAg-L but leads to stagnant HDV assembly in the cytoplasm [22]. Overexpression of GFP-CHC(1–107) produced as a dominant negative form of endogenous CHC and inhibited the secretion of HDV-III VLPs from Huh7 cells (Fig. 3C), while GFP alone did not affect the VLP secretion. Accordingly, both the GFP-CHC(1–107)-F27A and GFP-CHC(1–107)-E33A mutants inhibited the secretion of HDV-III VLPs to a lesser extent, indicating that these two mutants do not function as dominant negative forms of endogenous CHC. Taken together, our data indicate that HDV-III HDAg-L binds directly to CHC, and F27A and E33A are central to the role of CHC in HDV-III particle production.

### A novel W box situated in the C terminus of HDV-III HDAg-L enables binding to CHC

**Fig. 4:**
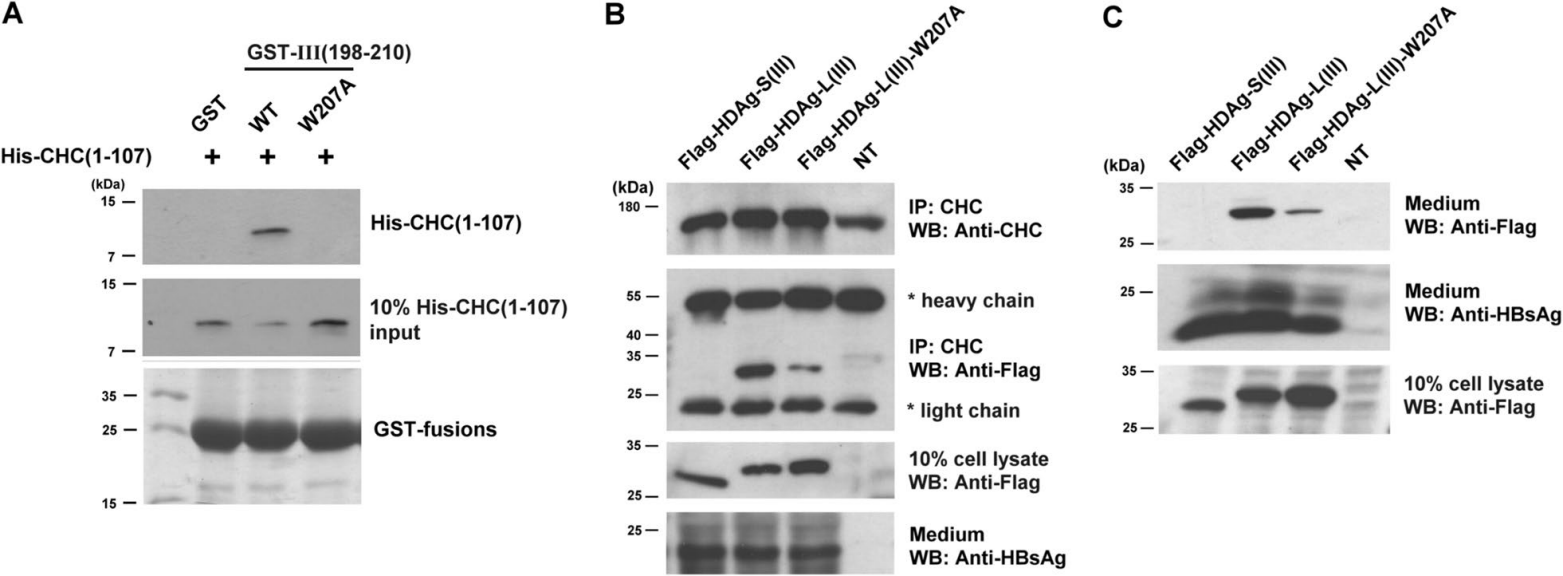
W207 situated in the C terminus of HDV-III HDAg-L enables binding to CHC. **A** Binding of His-CHC(1–107) to GST-III(198–210) or GST-III(198–210)-W207A. GST-tagged proteins were incubated with His-CHC(1–107). Levels of His-CHC(1–107) were determined by western blot analysis using anti-His antibody. Coomassie blue staining of GST fusion proteins is shown in the bottom panel. **B** Coimmunoprecipitation of CHC with HDV-III HDAg-L. The lysates of Huh7 cells cotransfected with plasmids pECE-C-ES and pCMV-Tag2C-HDAgS-III, pCMV-Tag2C-HDAgL-III, or pCMV-Tag2C-HDAgL-III-W207A were incubated with the anti-CHC antibody for precipitation. Levels of CHC, Flag-tagged HDAgs, and small HBsAg were determined by western blot analysis using anti-CHC, anti-Flag, and anti-HBsAg antibodies. **(C)** Secretion of HDV-III VLPs. HDV-III VLPs were harvested from the medium of Huh7 cells cotransfected with plasmids pECE-C-ES and pCMV-Tag2C-HDAgS-III, pCMV-Tag2C-HDAgL-III, or pCMV-Tag2C-HDAgL-III-W207A at day 4 posttransfection. Levels of Flag-tagged HDAgs and small HBsAg were determined by western blot analysis using anti-Flag and anti-HBsAg antibodies. Experiments were performed in triplicate

To identify the motif located at the C terminus of HDV-III HDAg-L which is responsible for clathrin-binding, GST fusion proteins of wild-type HDAg-L-III(198–210) and the HDAg-L-III(198–210)-W207A mutant were incubated with purified His-CHC(1–107) in comparable amounts. As shown in Fig. 4A, the GST-III(198–210)-W207A mutant failed to interact with His-CHC(1–107). In parallel, a coimmunoprecipitation assay was performed to investigate the interaction between CHC and wild type HDAg-L(III) or mutant HDAg-L(III)-W207A in Huh7 cells. Wild type HDAg-S(III) was used as the control. As shown in Fig. 4B, CHC interacted strongly with the wild-type HDV-III HDAg-L but associated weakly with the HDV-III HDAg-L-W207A mutant. In contrast, wild type HDAg-S(III) did not interact with CHC. The HDV-III HDAg-L-W207A mutant enabled the secretion of lesser amounts of HDV-III VLPs into the medium than the wild-type HDV-III HDAg-L (Fig. 4C). Taken together, mutation at W207 reduced the binding of HDV-III HDAg-L to CHC and, in turn, impeded the secretion of HDV-III VLPs.

### Sequence conservation of HDAg-L C-terminal sequences using phylogenetic analysis

**Fig. 5:**
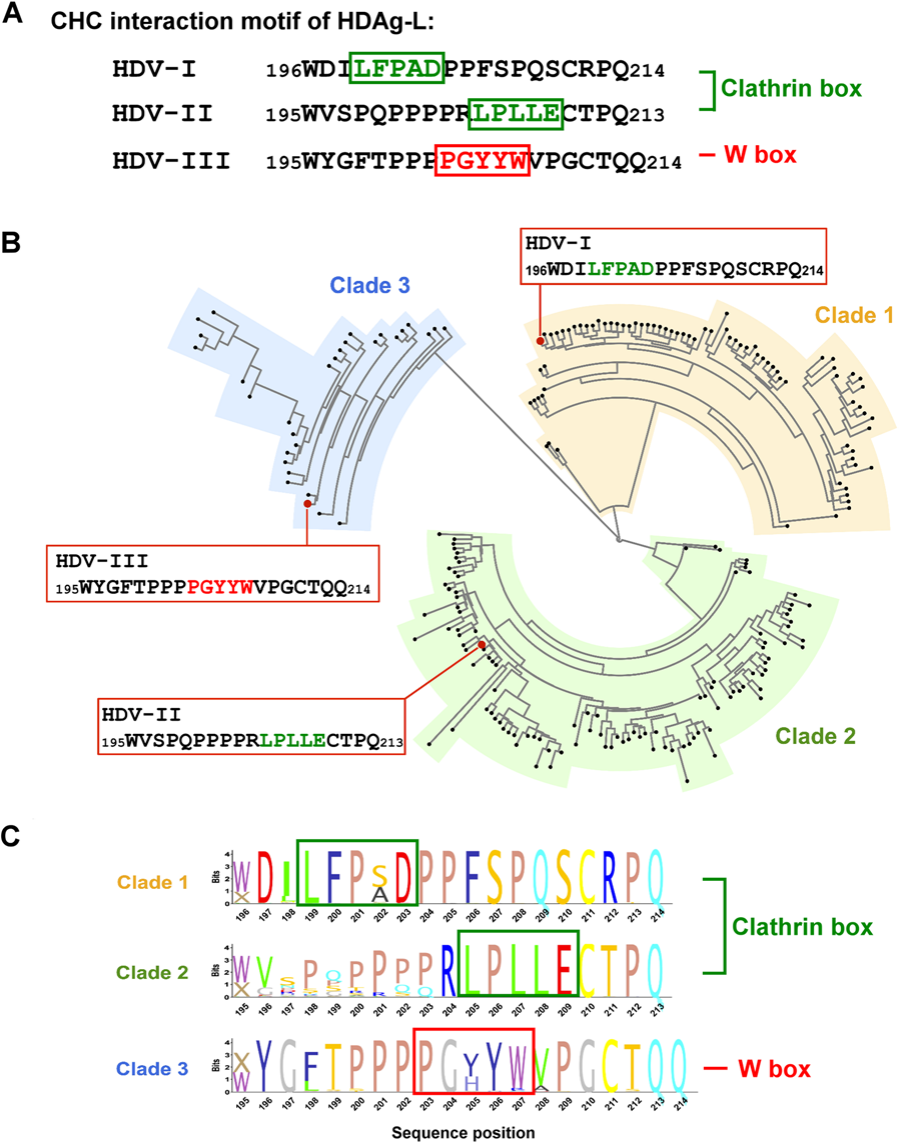
Identification of the novel W box at the C terminus of HDV-III HDAg-L. **A** The CHC interaction motifs of HDV-I, HDV-II, and HDV-III HDAg-L. The clathrin box (green rectangles) in HDV-I and -II HDAg-L, and the novel W box (red rectangle) in HDV-III HDAg-L are indicated. **B** Phylogenetic tree of HDAg-L. Unique C-terminal sequences of HDAg-L were analyzed using the neighbor joining method. The conventional HDV-I, -II, -III HDAg-L sequences (red dots) was clustered with other unique HDAg-L sequences (black dots) in clade 1 (shaded in orange), clade 2 (shaded in green), and in clade 3 (shaded in blue) as indicated. **C** Sequence logos of HDAg-L. The clathrin box (green rectangles) in clade 1 and 2 and the novel W box box (red rectangle) in clade 3 are indicated, respectively

A conserved clathrin adaptor sequence (clathrin box) in the C terminus of HDV-I HDAg-L, 199-LFPAD-203, and HDV-II HDAg-L, 205-LPLLE-209, has been proven to modulate the assembly of HDV viral particles, particularly residues L199 and D203 [21, 22]. Corresponding to the sequence diversity of the C terminus of HDAg-L, HDV-III HDAg-L contains a sequence motif 203-PGYYW-207 resembling a W box, rather than the conventional clathrin box, for CHC interaction (Fig. 5). To investigate amino acid sequence conservation of the C terminus of HDV-I, -II, and -III HDAg-L, 1628 accessions of HDAg-L were retrieved for phylogenetic analysis. As shown in Fig. 5B, three major clusters were identified, comprising 77, 110, and 30 unique sequences in clades 1, 2, and 3, respectively. Correspondingly, three conventional HDAg-L sequences of HDV-I, -II, and -III shown in Fig. 5A were resided in three different clades. Further, all the C-terminal sequences of HDAg-L were analyzed to determine the sequence conservation of clathrin box or W box in each clade. For clade 1, the C terminus of HDAg-L was highly conserved, identified as [W/X]DILFP[S/A]DPPFSPQSCRPQ (Fig. 5C). Although the C terminus of HDAg-L in clade 2 was relatively variable at positions 195–203, that at positions 204–213 was highly conserved containing the conventional clathrin box. On the other hand, the C terminus of HDAg-L in clade 3 was relatively conserved, identified as [X/W]YG[F/L]TPPPPG[Y/H]YWVPGCTQQ. Regarding the CHC interaction motif of HDAg-L, 199-LFP[S/A]D-203, 205-LPLLE-209, and 203-PG[Y/H]YW-207 were shared in clades 1, 2, and 3, respectively. Taken together, the clathrin box of HDV-I and -II HDAg-L, and the W box of HDV-III HDAg-L were highly conserved. In conclusion, our results highlight the indispensable role of a novel W box, present in HDV-III HDAg-L, for HDV-III viral particle formation.

## Discussion

Chronic coinfection with HBV and HDV is recognized as the most severe form of viral hepatitis, predisposing the onset of liver cirrhosis and hepatocellular carcinoma [3]. Accordingly, HDV infection is a major risk factor to human health. HDV-I and HDV-III infections lead to more adverse outcomes than HDV-II infection [28]. HDV-I is prevalent worldwide, and HDV-III is the most divergent genotype and has been linked to severe liver disease and fulminant hepatitis [2, 3]. It has been demonstrated in vitro that HDV-III is secreted more efficiently than HDV-I and HDV-II from Huh7 cells cotransfected with HBV genotype B, although the optimal combinations of HDV and HBV genotypes do not necessarily correlate with their geographic distribution [29]. In addition, another recent study raised the possibility that alternative viruses, not related to HBV, can also act as helper viruses for HDV, despite the fact that HDV is known as a satellite virus of HBV [1, 30]. In fact, the genotypes of HBV and HDV both are determinants for the severity of chronic hepatitis D [28].

In this study, we showed that HDV-I and HDV-III produced VLPs more efficiently from Huh7 cells expressing small HBsAg than did HDV-II. The direct interaction between CHC and the C terminus of HDAg-L differs among HDV genotypes, correlating with the VLP-forming ability of the particular HDV genotype. Corresponding to the results published previously, HDV-II VLPs in the medium are barely detectable in the presence of small HBsAg alone [22]. In addition, HDV-II HDAg-L demonstrated a reduced efficiency when forming HDV VLPs. The assembly efficiency of the various HDV genotypes correlates well with the ability of HDAg-L to interact with CHC. This may reflect the fact that there is lower pathogenicity among patients infected with HDV genotype II than among those infected with genotype I [5, 6]. The comparable levels of production of HDV-I and HDV-III VLPs from Huh7 cells expressing small HBsAg indicate that CHC binds with a similar efficiency to the clathrin box and the W box in the C-terminal regions of HDAg-L from HDV-I and HDV-III, respectively. Indeed, it has been shown that the dissociation constant of the canonical clathrin box is similar to that of the W box [15]. Furthermore, using site-directed mutagenesis, we confirmed that the C terminus of HDV-III HDAg-L contains a novel W box and that W207 is essential for the binding of HDAg-L to CHC.

Although long-term treatment with pegylated interferon-alpha (Peg-IFNα) may achieve a sustained virological response, most chronic HDV patients experience late viral relapses after treatment cessation [31]. Novel therapies targeting at critical steps in the HDV replication cycle are in development, specifically the entry (i.e., bulevirtide), assembly (i.e., lonafarnib), and export (i.e., REP-2139) of HDV particles [31]. In particular, lonafarnib prevents the post-translational prenylation of HDAg-L by inhibiting the host farnesyl transferase activity [31] and shows pan-genotypic activity against HDV infection in vitro [29]. We have shown previously that the TAT-HA-HDAg-L(198–210) fusion protein inhibits HDV-I infection by competing with the C terminus of HDV-I HDAg-L for interaction with CHC [32]. In our previous study, we discovered the conventional clathrin box of HDV-I, and -II HDAg-L [21, 22]. However, no conventional clathrin box sequence was identified in HDV-III HDAg-L. In this study, we demonstrated that HDV-III HDAg-L contains a novel W-box at the C terminus, and that its presence is responsible for clathrin interaction and viral assembly. According to our finding that clathrin binding has a major role in the assembly and secretion of HDV-III, inhibiting the association of CHC and HDV-III HDAg-L may also be a plausible therapeutic approach.

## Conclusion

In the present study, we showed that the C terminus of HDV-III HDAg-L contains a novel clathrin binding sequence that associates with the W box-binding residues of CHC. In addition, post-Golgi vesicular trafficking enables the secretion of HDV-III VLPs. Understanding the alternative clathrin binding mechanism in HDV infection not only helps clarify the mode of assembly of HDV-III VLPs but also emphasizes the importance of clathrin binding in the virus assembly of the various HDV genotypes. Clathrin is an alternative molecular target that has therapeutic potential for HDV management. Revealing the key role of CHC in HDV infection not only facilitates the understanding of HDV morphogenesis and pathogenesis but also may aid the management of viral hepatitis in the future.

## Supplementary Information

**Additional file 1. Fig. S1.** Binding of HDV-I, HDV-II, and HDV-III HDAg-L to CHC.

**Additional file 2. Table S1.** HDAg-L C-terminal sequences retrieved from NCBI using taxid:12475 (HDV).

**Additional file 3. Table S2.** Unique HDAg-L C-terminal sequences in the phylogenetic tree and the corresponding NCBI accessions.

## Data Availability

All data generated or analysed during this study are included in this published article.

## Abbreviations

HDV: Hepatitis delta virus
HDAg-L: Large hepatitis delta antigens
CHC: Clathrin heavy chain
VLPs: Virus-like particles
CCVs: Clathrin-coated vesicles
HBV: Hepatitis B virus
HBsAg: Hepatitis B surface antigen
PTMs: Post-translational modifications
RNP: Ribonucleoprotein
TGN: *trans*-Golgi network
