# Supplementary material for: Identification of a novel interaction site between the large hepatitis delta antigen and clathrin that regulates the assembly of genotype III hepatitis delta virus
Source: Virol J. 2022 Oct 17;19:163. doi: 10.1186/s12985-022-01866-3 (PMC9578201; doi:10.1186/s12985-022-01866-3)
Supplement: Supplementary file 1 — Additional file 1. Fig. S1. Binding of HDV-I, HDV-II, and HDV-III HDAg-L to CHC. [file 12985_2022_1866_MOESM1_ESM.docx]

**Additional File 1: Fig. S1. Binding of HDV-I, HDV-II, and HDV-III HDAg-L to CHC.**


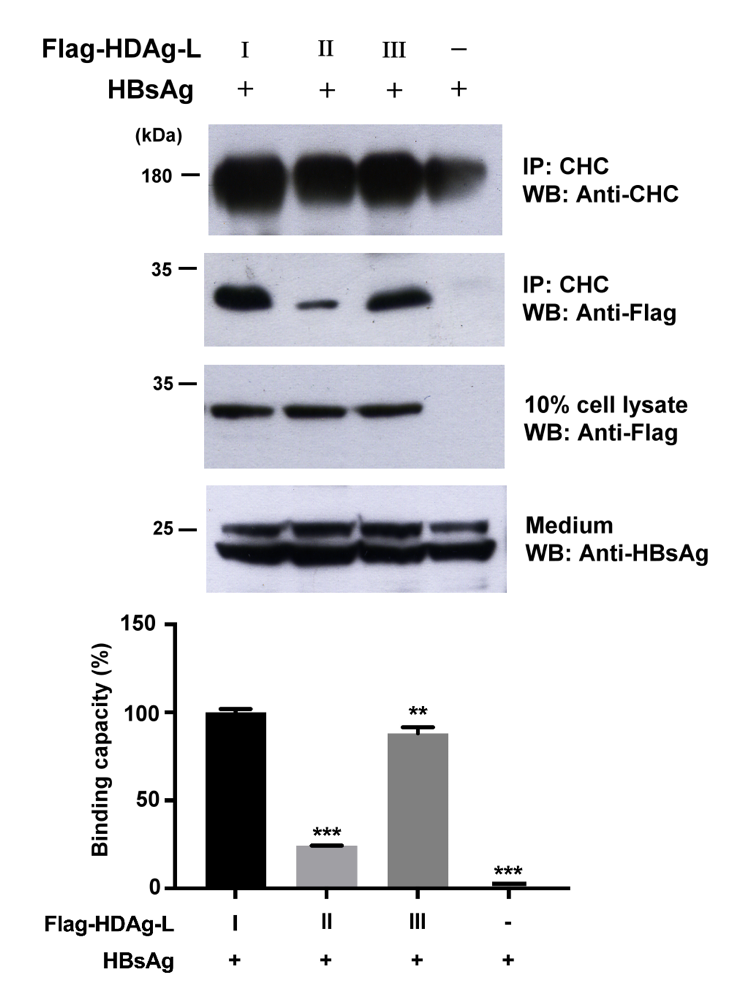


**Fig. S1. Binding of HDV-I, HDV-II, and HDV-III HDAg-L to CHC.** Coimmunoprecipitation of CHC with Flag-tagged HDAg-L. Lysates of COS7 cells cotransfected with plasmids encoding HBsAg and Flag-tagged HDV-I, HDV-II, or HDV-III HDAg-L were incubated with the anti-CHC antibody for precipitation. Levels of CHC, Flag-tagged HDAg-L, and small HBsAg were determined by western blot analysis by using anti-CHC, anti-Flag and anti-HBsAg antibodies. Experiments were performed in triplicate. Results of western blots were quantified by densitometry analysis.
